# The effect of respiratory metaboreflex on cerebral haemodynamics during exercise

**DOI:** 10.1113/EP093410

**Published:** 2025-11-03

**Authors:** Rasmus Kopp Hansen, Niels H. Secher, Markus Wendt Schelske, Stefanos Volianitis

**Affiliations:** ^1^ Respiratory and Critical Care Group, Department of Health Science and Technology Faculty of Medicine, Aalborg University Aalborg Denmark; ^2^ Department of Anesthesia, Rigshospitalet, Department of Clinical Medicine University of Copenhagen Copenhagen Denmark; ^3^ Department of Clinical Medicine Rigshospitalet Copenhagen Denmark; ^4^ GUR Aalborg Denmark; ^5^ Department of Sport Coaching College of Sport Sciences, Qatar University Doha Qatar

1

Dear Editor,

We read the paper by Ramsook et al. (2026) with great interest because it addresses an important gap in our understanding of cardiovascular consequences of the respiratory muscle metaboreflex for cerebral blood flow (CBF), with implications for the debated sympathetic control of the brain circulation (Brassard et al., 2017).

The study investigated cerebrovascular haemodynamic responses to high‐intensity fatiguing inspiratory muscle work applied with inspiratory pressure threshold loading, designed to provoke the respiratory metaboreflex and, based on the lack of significant changes on cerebrovascular conductance, concluded that cerebrovascular conductance is prioritized and less likely to be impacted by systemic alterations in cardiovascular function. These findings provide support to the notion that sympathetic activity has limited control of the brain circulation.

However, the contribution of the respiratory metaboreflex in the redistribution of cardiac output is manifested during sustained high‐intensity whole‐body exercise, in which the associated elevated ventilation provokes diaphragmatic fatigue and limits blood flow to active muscles (Harms et al., 1997). As acknowledged by the authors, the effect of the respiratory metaboreflex is likely to be more pronounced during high‐intensity large‐muscle‐mass exercise, which introduces competition between vascular beds for the available cardiac output and, therefore, its effect on cerebral haemodynamics might be investigated more directly. This question was addressed by Hansen et al. (2020), who used isocapnic end‐tidal forcing to eliminate the effect of CO_2_ on CBF during sustained high‐intensity (≥90% of maximal oxygen uptake) cycling to exhaustion (∼12.5 min) that induced inspiratory muscle fatigue. Interestingly, during the isocapnic cycling trial, which removed the influence of CO_2_, CBF was not only maintained but it even increased in the final  min, demonstrating a dissociation from cerebral oxygenation, which continued to decline. These findings are seemingly in agreement with the conclusion by Ramsook et al. (2026) and can be interpreted as a lack of appreciable sympathetic vasoconstrictive consequence of the respiratory metaboreflex on the cerebral vasculature during exercise. However, considering the multitude of factors affecting the regional CBF, it cannot be excluded that the increased CBF in the final  min of exercise is a manifestation of elevated neural activation overriding any potential sympathetic influence.

Although we accept the obvious importance of CBF for cognitive function etc., it nevertheless seems that in order to establish a systemic blood flow distribution ‘hierarchy’, the plethora of interactive CBF determinants during exercise, which differ greatly from those at rest, need to be addressed.
